# Supplementary material for: Sustained-input switches for transcription factors and microRNAs are central building blocks of eukaryotic gene circuits
Source: Genome Biol. 2013 Aug 23;14(8):R85. doi: 10.1186/gb-2013-14-8-r85 (PMC4054853; doi:10.1186/gb-2013-14-8-r85)
Supplement: Additional file 5 — HTML Browsable Motif Output. Zipped folder containing all WaRSwap and FANMOD motif output, viewable in a web browser. [file gb-2013-14-8-r85-S5.ZIP › HTML_browsable_motif_output/WaRSwap_all_tair9/sigs_WSWRSLDraw-2000_all.pvals.heatmaps.html/motif_id_166_010100110_tftype_all_upstream_-1000_0.html]

```
BG_MODEL = Rnew2
MOTIF_ID = 166_010100110
TF_TYPE = all
UPSTREAM = -1000_0


PVals
FN_0.2	FN_0.4	FN_0.6	FN_0.8
dg_60.genes	0.739	0.001	0.03	0.051
dg_70.genes	0.748	0.002	0.041	0.05
dg_80.genes	0.735	0.002	0.043	0.044

ZScores
FN_0.2	FN_0.4	FN_0.6	FN_0.8
dg_60.genes	-0.651	2.883	1.935	1.845
dg_70.genes	-0.689	2.9	1.852	1.893
dg_80.genes	-0.634	2.877	1.846	1.946

StDevs
FN_0.2	FN_0.4	FN_0.6	FN_0.8
dg_60.genes	1220.204	286.295	52.684	6.484
dg_70.genes	1221.896	290.141	54.816	6.495
dg_80.genes	1209.36	287.811	54.688	6.245
```
